# Tumor cells escape immunosurveillance by hampering LFA-1

**DOI:** 10.3389/fimmu.2025.1519841

**Published:** 2025-01-22

**Authors:** Shishir Upadhyay, Lewis Murugu, Lena Svensson

**Affiliations:** ^1^ Department of Molecular Biology, Umeå University, Umeå, Sweden; ^2^ Umeå Centre for Microbial Research, Umeå University, Umeå, Sweden

**Keywords:** LFA-1, leukocytes, cancer, TME, immunosurveillance, immune escape

## Abstract

During tumor immunosurveillance, leukocytes play a crucial role in the cellular defense system, working collaboratively with other immune components to recognize and eliminate aberrant cells. Integral to this process is the integrin Lymphocyte Function-Associated Antigen 1 (LFA-1). LFA-1 facilitates adhesion during leukocyte migration and helps establish stable cell-to-cell contacts between leukocytes and their targets. Additionally, as a receptor, LFA-1 signaling activates leukocytes, promoting their differentiation and effector functions against cancer. However, tumors can develop mechanisms to evade immune clearance by disrupting LFA-1 functions or hijacking its pathways. In this review, we first detail how leukocytes utilize LFA-1 during immunosurveillance and then explore how tumors counteract this process in the tumor microenvironment (TME) by either altering LFA-1 functions or exploiting it to drive tumorigenesis. Moreover, we discuss therapeutic strategies targeting LFA-1, including inhibitors tested in laboratory studies and animal models, highlighting their potential as anticancer interventions and the need for further research to evaluate their clinical utility.

## 1 Introduction

Leukocytes serve as the immune system’s primary cellular defense against cancer, performing distinct roles across the immune response, from tumor recognition to destruction. To execute these functions effectively, leukocytes depend heavily on integrins. Integrins are transmembrane receptors composed of alpha and beta subunits that assemble into heterodimers essential for cell adhesion and signal transduction (1–4). Among the integrins expressed in leukocytes, several, including αLβ2, αMβ2, αXβ2, αDβ2, α4β1, α4β7, and αEβ7, have been characterized and extensively reviewed elsewhere (1, 2, 5, 6). Of these, αLβ2, or Lymphocyte Function-Associated Antigen 1 (LFA-1), is a key leukocyte-specific integrin predominantly expressed in lymphocytes, with lower levels in other leukocytes (7–13). Due to its essential roles in leukocyte function, LFA-1 is the primary focus of our research and this review.

LFA-1 is pivotal to the immune system, mediating the migration and effector functions of leukocytes at sites of infection and inflammation. This function is primarily facilitated through its interaction with ICAM-1, its main ligand (8, 14). However, beyond ICAM-1, LFA-1 can bind to other ligands, including ICAM-2, ICAM-3, ICAM-4, ICAM-5, and JAM-A, which are expressed on various cell types such as endothelial cells and immune cells (15–19). These interactions are critical during the immune response and have been discussed in greater detail in the literature (15–22).

In T cells, LFA-1 supports adhesion to the vascular endothelium, enabling their exit from the bloodstream and subsequent entry into tissues (23). It also guides T cells into lymph nodes through high endothelial venules (HEVs) and plays an essential role in forming immunological synapses with antigen-presenting cells (APCs), a key step in initiating the adaptive immune response (24, 25). In cytotoxic T cells, LFA-1 stabilizes contact with infected or abnormal cells, including tumor cells, thereby enhancing their ability to eliminate these targets. For LFA-1 to function, it must transition from an inactive, bent-closed conformation to an active, extended-open state capable of ligand binding. This activation is mainly driven by inside-out signaling, initiated by signals from other transmembrane proteins such as chemokine receptors, T cell receptors, and selectin ligands (1, 3, 26, 27). Inside-out signaling involves the activation and recruitment of RAP-1, which facilitates the association of RAPL/MST1 with the LFA-1 αL subunit (28–31). RAP-1 and RIAM further recruit talin to the LFA-1 β chain, where talin, in conjunction with kindlin-3, stabilizes the integrin in its high-affinity state, promoting effective ligand binding and initiating outside-in signaling cascades that drive T cell adhesion and migration (32–34). Interestingly, recent findings from Springer’s group suggest that ligand binding itself may induce the conformational change from the bent-closed to the extended-open form, potentially playing a more significant role than talin in stabilizing this state (35). This challenges the previously understood sequence, implying that outside-in signaling may precede and facilitate inside-out activation (35, 36).

Although LFA-1 is a major integrin for T cells, it also plays a role in the function of other leukocyte subsets. For instance, LFA-1 facilitates natural killer (NK) cell activation and the formation of cytotoxic immune synapses with target cells (37–40). Additionally, it facilitates dendritic cell (DC) transmigration from tissues into afferent lymphatic vessels (41), and supports the migration and endothelial patrolling of non-classical monocytes (42), as observed in murine models. Given its central role in the optimal functioning of lymphocytes, particularly T cells and NK cells, LFA-1 is indispensable for effective tumor immunosurveillance. However, as cancer progresses, tumor cells can adopt mechanisms to evade immune detection (43–45). One such strategy involves disrupting LFA-1 function, thereby compromising the ability of leukocytes to mount an effective defense. Notably, impaired activation or conformation of LFA-1 has been linked to certain forms of cancer (46, 47).

This review paper will examine how modulation of LFA-1 in the tumor microenvironment (TME) contributes to immune evasion and tumor progression. We will first describe how LFA-1 generally promotes immune surveillance through its interactions with ligands, primarily its major ligand, ICAM-1, leading to leukocyte activation and tumor cell killing. Next, we will broadly outline three tumor immune-escape strategies involving LFA-1: altered LFA-1-mediated migration and infiltration of leukocytes into the tumor microenvironment, disrupted LFA-1 functionality in leukocytes within the tumor microenvironment, and LFA-1-mediated tumor invasiveness. These mechanisms not only highlight the multifaceted role of LFA-1 in tumor biology but also represent potential targets for immunotherapeutic interventions. In this context, we will discuss inhibitors and drugs, evaluated in *in vitro* and *in vivo* systems, that target various stages of LFA-1 modulation and show potential as anticancer therapies.

## 2 LFA-1 mediates antitumor immunosurveillance

**Figure 1:**
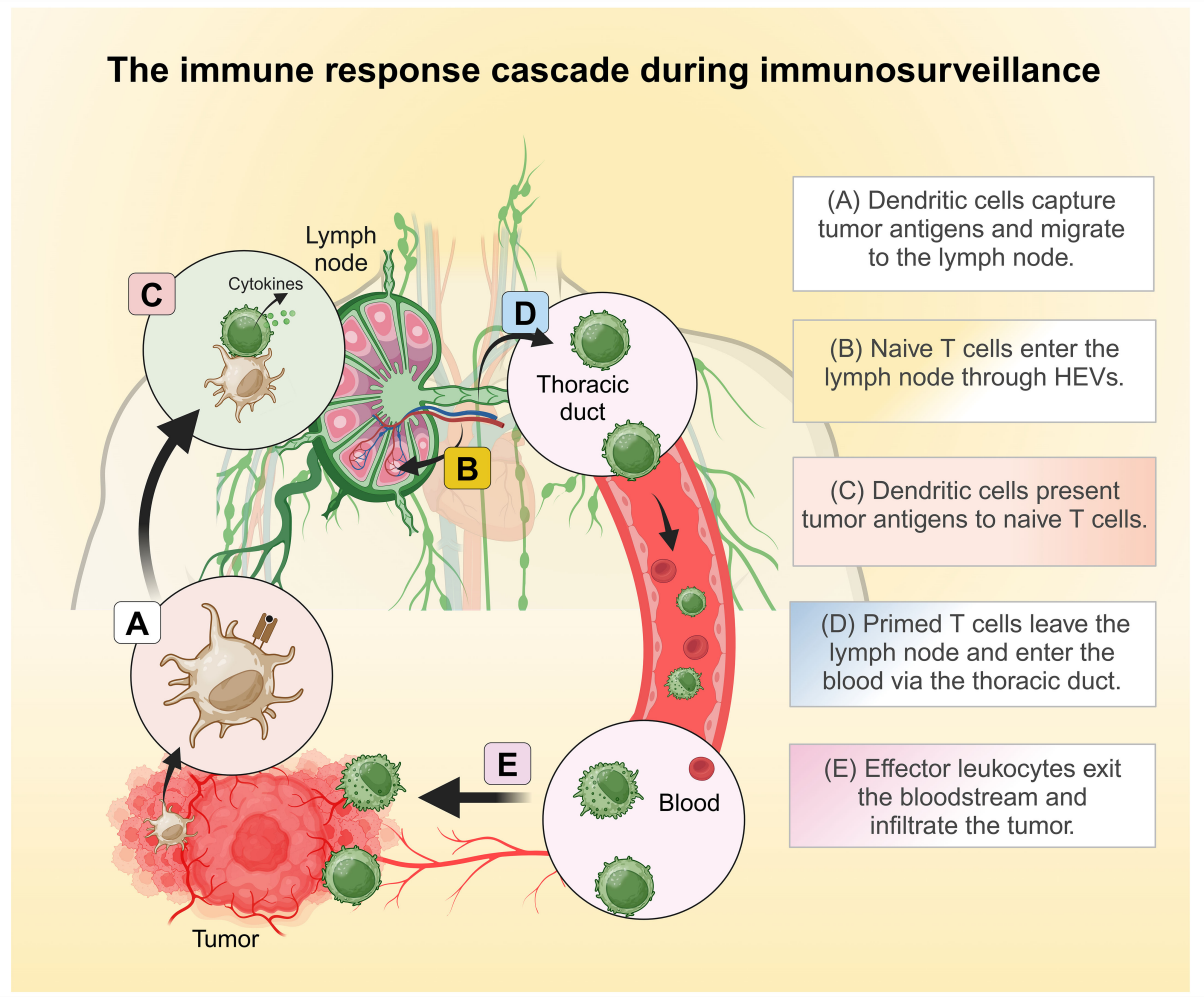
The general immune response cascades during immunosurveillance. **(A)** Dendritic cells capture tumor antigens and shuttle to the draining lymph nodes. **(B)** Naïve T cells enter the lymph nodes through high endothelial venules (HEVs). **(C)** In the lymph nodes, dendritic cells present tumor antigens to naïve T cells, thereby initiating T cell activation. **(D)** Primed T cells leave the lymph nodes and enter circulation via the thoracic duct. **(E)** Effector leukocytes exit the bloodstream and infiltrate the tumor. Created in BioRender. Murugu, L. (2024) https://BioRender.com/f91b170.

**Figure 2:**
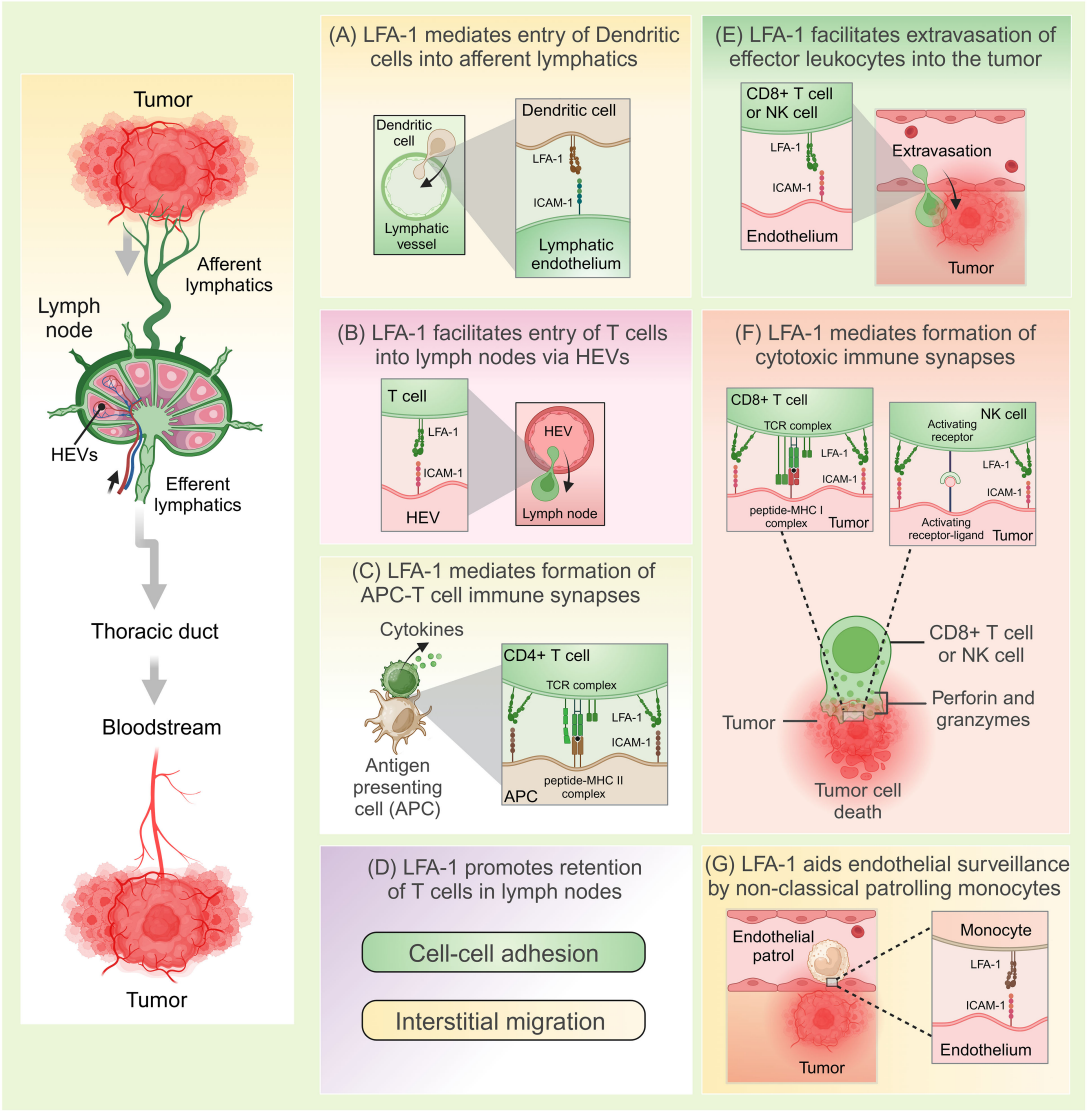
LFA-1-mediated anti-tumor immunosurveillance. **(A)** LFA-1 interacts with ICAM-1 to facilitate entry of dendritic cells from the tumor sites into the afferent lymphatics. **(B)** LFA-1 mediates migration of naïve T cells into the lymph nodes via high endothelial venules (HEVs) through its interactions with ICAM-1. **(C)** LFA-1 mediates formation of immunological synapses between antigen presenting cells (APCs) such as dendritic cells and T cells. Following antigen presentation, T cells are activated and release cytokines. **(D)** Through its interactions with ICAM-1, LFA-1 promotes retention of T cells in the lymph nodes optimizing their chances of encountering cognate antigens. This happens through efficient interstitial migration and formation of cell-to-cell attachments that function to delay T cell egression. **(E)** LFA-1 facilitates extravasation of effector leukocytes into the tumor. **(F)** LFA-1 mediates formation of cytolytic immune synapses and the eventual killing of tumors. **(G)** LFA-1 aids endovascular surveillance by patrolling non-classical monocytes. Created in BioRender. Murugu, L. (2024) https://BioRender.com/g87c843.

Tumor cells often display abnormal or mutated proteins, known as tumor-associated antigens (TAAs), which are absent in healthy cells. These TAAs are recognized as foreign by the body’s defense mechanisms, initiating a process called immunosurveillance (43, 45). This process involves a coordinated series of events where innate immune cells detect TAAs, eventually triggering the activation, migration, and infiltration of tumor-specific cytotoxic lymphocytes into the tumor microenvironment. Once within the TME, these lymphocytes identify and destroy tumor cells by releasing cytotoxic mediators. LFA-1 plays a pivotal role throughout this antitumor immune response, facilitating critical steps such as immune cell activation, adhesion, migration, and target cell elimination. This section explores how immunocompetent cells rely on LFA-1 for effective tumor eradication. **Figure 1** provides a general overview of the immune response cascade, while **Figure 2** emphasizes the specific stages where LFA-1 is indispensable.

### 2.1 Recognition of tumor associated antigens in the TME

The initial step in immunosurveillance is the recognition of tumor associated antigens in the TME. Dendritic cells (DCs) play a key role in this process. After engulfing TAAs, DCs process and present these peptides on their surface MHC molecules before migrating to draining lymph nodes to function as antigen-presenting cells (APCs) (44). The migratory process is initiated by chemotaxis and is guided by the CCR7 receptor on DCs, which binds to CCL21—a ligand constitutively expressed by lymphatic endothelial cells. Ongoing inflammation in the TME releases TNF-α, which further elevates CCL21 levels. This increase in CCL21 in turn, promotes LFA-1 activation in DCs, mediating their transendothelial migration into the afferent lymphatic vessels, ultimately guiding them to the draining lymph nodes (41).

### 2.2 Priming of T cells in the draining lymph nodes

Once in the lymph nodes, DCs orchestrate an effective antitumor response by priming and activating naïve tumor-specific CD4+ and CD8+ T cells. These naïve T cells rely on LFA-1 to migrate and enter the lymph nodes through HEVs. Chemokines CCL19 and CCL21 from the lymph nodes as well as within HEVs, facilitate T cell migration by binding to CCR7 on T cells, triggering inside-out signaling that activates LFA-1. Once activated, LFA-1 on T cells binds to ICAM-1 on HEVs enabling their transendothelial entry into the lymph nodes (19). Within the lymph nodes, LFA-1 further aids effective antigen scanning by T cells, helping them locate their cognate antigen presented by APCs (48). Here, the adhesive state of LFA-1 in T cells is regulated by bidirectional signaling—inside-out signaling adjusts its affinity, and outside-in signaling provides feedback from LFA-1 engagement (49, 50). This dual signaling mechanism modulates T cell adhesion, ensuring that T cells migrate effectively within lymphoid tissue, optimizing their surveillance and interactions with tumor antigens. Notably, unlike chemokine-induced inside-out signaling, which alone can activate LFA-1 to a high-affinity state in T cells, full LFA-1 activation through TCR signaling requires LFA-1 to bind to ICAM-1 on APCs (51). In DCs, however, regulation of LFA-1’s activity plays a key role in DC/T cell conjugate formation, with mature DCs expressing elevated levels of cytohesin-1–interacting protein (CYTIP) to maintain LFA-1 in an inactive state (48). This inactivity is essential for the release of T cells and their subsequent priming. Additionally, the lower lateral mobility of ICAM-1 on mature DCs compared to immature DCs increases the likelihood of binding between LFA-1 on naïve T cells and ICAM-1 on mature DCs (52). The strength of LFA-1 binding to ICAM-1 on DCs, combined with CD2/CD58 interactions, improves T cell antigen discrimination (53). This process likely ensures that T cells bind specifically to non-self-antigens, including tumor antigens, through their TCR. Furthermore, LFA-1 binding to ICAM-1 within the lymphoid parenchyma increases T cell retention in the lymph nodes, preventing their immediate exit and allowing more time for T cells to encounter their cognate antigen (54). While our review paper focuses on the role of integrins, it is important to note that interstitial migration of leukocytes within lymph nodes may occur independently of integrins, as has been extensively demonstrated in other studies (55–58).

*In vitro* studies indicate that LFA-1/ICAM-1 interactions may promote CD4+ T cell differentiation towards the Th1 phenotype (59). Th1 cells are crucial for tumor elimination as they produce TNF-α and IFN-γ, which stimulate the recruitment and activation of cytotoxic CD8+ T cells (CTLs), NK cells, and monocytes to the tumor microenvironment, while also negatively regulating tumor-promoting Th17 cells (60). CD4+ T cells further support CD8+ T cell expansion by forming LFA-1-dependent transient homotypic synapses with adjacent activating CD8+ T cells (61, 62). Following differentiation, tumor-specific effector T cells exit the lymph nodes and migrate to peripheral tissues to perform their immune functions.

Unlike T cells though, NK cells can directly identify tumor cells without prior priming by APCs. NK cells become “educated,” meaning they gain the ability to recognize tumor cells during their development. Mature NK cells are characterized by high LFA-1 expression, as well as the expression of receptors such as NKp46, NKG2D, CD16, and elevated levels of perforin and granzyme B (63, 64). LFA-1, along with other cell-surface adhesion receptors including Mac-1, regulate the priming of NK cells and facilitates their homing and migration to both lymphoid and non-lymphoid organs (65, 66).

### 2.3 Entry and action of effector leukocytes in the TME

T cells exit the lymph nodes via the efferent lymphatic vessels and drain into the bloodstream through the thoracic duct (67). Their exit from the bloodstream into the TME is dependent on the interaction between LFA-1 and ICAM-1 on endothelial cells. A recent study in mice models highlighted the intriguing influence of the circadian clock on the transmigration of CTLs into tumors, with increased ICAM-1 expression on endothelial cells being observed in the morning, thereby enhancing T cell infiltration during this time (68). Similarly, NK cells are recruited to the tumor site through inflammation-induced signaling in response to interleukins (IL-12, IL-15, and IL-18) from target cells, with LFA-1 playing a crucial role in their migration (69, 70).

In addition, tumor-associated HEVs exhibit elevated ICAM-1 expression (71, 72), which may further facilitate lymphocyte infiltration into tumors and support the formation of tertiary lymphoid structures. Notably, a study of resected primary human breast carcinomas revealed that the density of ICAM-1-expressing HEVs predicted both the extent of T and B cell infiltration and clinical outcomes in breast cancer (72).

Within the TME, migrated CTLs recruit LFA-1 to engage with ICAM-1 on tumor cells, forming a cytolytic synapse ultrastructure that facilitates their effector functions. This includes the directed delivery of cytotoxic granules, which contain granzymes and perforin, through secretory clefts of the mature lytic synapse. The efficient delivery of these cytolytic granules depends on the arrangement of the microtubule-organizing center (MTOC), which is also influenced by Mg^2+^ and Ca^2+^ (73–75). Importantly, the conformational activation of LFA-1 is essential for licensing the exocytosis process, which enhances the local concentration of cytotoxic molecules to optimize tumor cell recognition and potentiate the antitumor immune response (74). What is more, earlier studies have underscored the importance of functional CTLs in antitumor responses. In particular, a study involving LFA-1-deficient mice demonstrated that these mice were unable to clear tumors injected into their footpads, which was attributed to a defective CTL response against the tumor cells (76). LFA-1 is also crucial for the mechanosensitive recognition of tumor cells by NK cells and facilitates the formation of lytic synapses between them (65). Upon successful synapse formation, NK cells release lytic granules, including perforin and granzymes (77, 78). Perforin creates pores in the target cell membrane, allowing granzymes to enter and trigger apoptosis, effectively killing tumor cells. The efficiency of NK cell degranulation and cytotoxicity is influenced by LFA-1 and a high Ca^2+^ influx (79). Moreover, activation with IL-2 further enhances NK cells’ broad antitumor cytotoxicity. NK cells can also destroy tumors through Antibody-Dependent Cellular Cytotoxicity (ADCC), where Fc receptor-bearing NK cells recognize and kill antibody-coated tumor cells. However, in the absence of LFA-1, NK cells are unable to effectively destroy their targets. A study of four patients with LFA-1 deficiency supports this, showing that their NK cells exhibited impaired cytotoxicity in cellular assays, despite retaining functional Fc receptors (80).

From a functional perspective, the role of LFA-1 in mediating immune surveillance is heavily dependent on its proper activation within T cells. Several proteins and factors regulate LFA-1 activation, conformational changes, and functions. For instance, layilin, a membrane glycoprotein expressed by certain tumor-specific CTLs, enhances LFA-1 activation by stabilizing its interaction with ICAM-1. Together with talin, layilin supports LFA-1-mediated adhesiveness and the cytotoxic activity of CTLs, playing a critical role in promoting immune synapse formation and tumor cell killing, as demonstrated in melanoma (81). However, further research is needed to fully elucidate the layilin-mediated pathways affecting LFA-1 expression and activation before these findings can be applied to therapeutic interventions. Also, certain co-receptors, such as CD226 (DNAM-1), regulate LFA-1 function by enhancing its adhesive properties through the regulation of its affinity conformation. Evidence shows that the loss of CD226 impairs LFA-1’s high-affinity conformation in CTLs, a change associated with malignancy, including multiple myeloma and lung adenocarcinoma (82). In CLL, T cells exhibit significantly reduced LFA-1 expression or impaired LFA-1 function, leading to their failure to adhere to and transmigrate across VCAM-1, ICAM-1, and CXCL12-expressing endothelium, which results in diminished trafficking to lymph nodes (83, 84). Taken together, it is apparent that proper functioning of LFA-1 is crucial for optimal antitumor CTL responses during immunosurveillance.

The role of LFA-1 in mediating immune surveillance against cancer by innate immune cells beyond NK cells remains underexplored. Nevertheless, some studies suggest that certain tumors are targeted by specific subsets of monocytes and neutrophils, despite differences in LFA-1 regulation and activation between these cell types (85). For example, through intravital microscopy studies in mice, non-classical monocytes have been shown to patrol the vasculature and maintain endothelial homeostasis by scavenging microparticles (42). This patrolling behaviour is facilitated by the expression of LFA-1 on these cells, enabling interactions with ICAM-1-rich endothelial cells (42). In lung cancer mouse models, these “intravascular housekeepers” have been observed removing metastatic tumor cells from vascularized tissues (86). Upon detecting primary tumor invasion, non-classical monocytes decrease their patrolling activity, migrate toward tumor particles, and engulf them (86). Similarly, tumor-entrained neutrophils (TENs) can suppress metastatic seeding in the premetastatic lung in mice underscoring their roles in limiting tumor spread (87). In addition, ICAM-1 expression on breast cancer cells in mice facilitates interactions with neutrophils that suppress lung metastasis, independent of cytotoxic T cell activity (88).

## 3 LFA-1 functions in engineered T cells

LFA-1 and its interaction with ICAM-1 have essential roles in the antitumor responses of Chimeric Antigen Receptor (CAR) T cells, much like their involvement in CTLs (89–91). CAR T cells are engineered from allogenic T cells, genetically modified to express a Chimeric Antigen Receptor (CAR) that recognizes the patient’s tumor-associated antigens (89, 90). These cells are expanded *in vitro* and infused back into the patient.

The LFA-1-ICAM-1 axis is particularly important for CAR T cell function, as evidenced by the differential effectiveness of CAR T cells in hematological versus solid tumors (74, 89–95). In solid tumors, CAR T cells often struggle to migrate to the tumor site and instead accumulate in non-target tissues. This phenomenon necessitates the infusion of large quantities of highly activated T cells, which raises both the risk of T cell toxicity and systemic cytokine release syndrome (CRS) while also significantly increasing the manufacturing costs of CAR T cell therapies (89). To better understand why *in vitro*-activated CD8+ T cells become sequestered in off-target tissue sites following intravenous transfer, Hong et al. used intravital microscopy in mice and a CRISPR-Cas9 screen, discovering that ST3GAL1-mediated glycosylation of CD18 disrupts LFA-1 recycling, leading to nonspecific sequestration of T cells in off-target tissues (93). Enhancing the expression of βII-spectrin in the CAR T cells counteracted this effect, improving tumor-specific homing and reduced tumor growth in mice. These findings suggest that targeting the ST3GAL1–βII-spectrin axis could enhance CAR T cell therapies by improving migration to tumor sites (93).

Moreover, ICAM-1 expression on tumor cells is critical for CAR T-cell functionality (94, 95). A CRISPR-based screen in a multiple myeloma cell line revealed that knockout of the ICAM-1 gene in tumor cells led to resistance to B-cell maturation antigen (BCMA) CAR T cells (95). Conversely, increased expression of ICAM-1 on target tissues has been identified as a limiting factor for CAR T cell efficacy. In one study, ICAM-1 expression was significantly associated with advanced stages of gastric cancer and poorer survival rates in human patients (94). Furthermore, ICAM-1-targeting CAR T cells exhibited substantial efficacy *in vitro*, with their effectiveness correlating to the level of ICAM-1 expression in target cells. In animal models, these CAR T cells successfully eliminated lung tumors but were less effective against peritoneal tumors. To enhance their efficacy, the study explored combinations with paclitaxel or CAR activation-dependent IL-12 release, which significantly increased anti-tumor activity and improved survival (94). These observations suggest that ICAM-1-targeting CAR T cells, alone or combined with chemotherapy, could be a promising strategy for treating patients with ICAM-1 positive advanced gastric cancer.

Another major challenge hindering CAR T cell efficacy is their limited ability to effectively leverage accessory receptors like LFA-1. A study demonstrated that CAR T cells exhibit over 100-fold lower antigen sensitivity than TCRs when antigens are presented on APCs, a disparity not observed with purified proteins (96). This is because CARs inefficiently utilize receptors such as CD2 and LFA-1, which significantly enhance TCR sensitivity but have minimal impact on CARs. Engineering approaches, such as fusing CARs to the TCR CD3ϵ subunit (TRuCs) or incorporating TCR αβ variable domains (STARs/HITs), restored sensitivity by improving accessory receptor engagement (96). These findings highlight the potential of enhancing CAR T cells’ interaction with LFA-1 to improve their therapeutic efficacy.

Together, these studies underscore the critical role of LFA-1 in CAR T cell efficacy and suggest that overcoming barriers related to migration, ICAM-1 expression, and receptor utilization could significantly enhance CAR T therapies, particularly in solid tumors.

## 4 Tumors escape immunosurveillance by altering LFA-1-mediated functions

**Figure 3:**
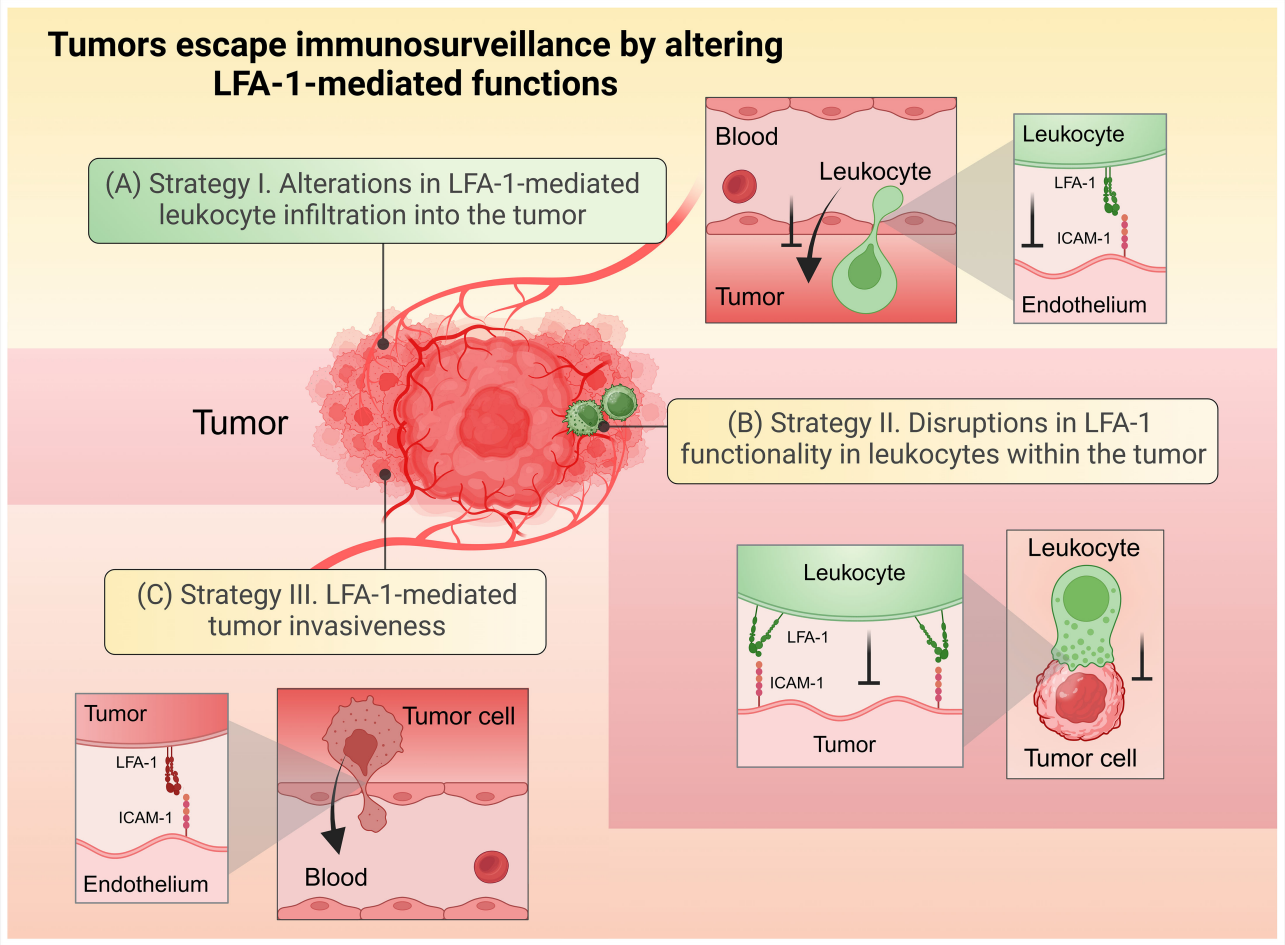
Tumors escape immunosurveillance by altering LFA-1-mediated functions. **(A)** Strategy (I) Alterations in LFA-1-mediated leukocyte infiltration into the tumor. **(B)** Strategy II. Disruptions in LFA-1 functionality in leukocytes within the tumor environment. **(C)** Strategy III. LFA-1-mediated tumor invasiveness. Created in BioRender. Murugu, L. (2024) https://BioRender.com/z47w979.

Tumors employ several strategies to evade immune clearance by disrupting LFA-1-mediated processes. As broadly depicted in **Figure 3**, these disruptions generally affect anti-tumor immune functions by impairing leukocyte adhesion to tumor vasculature, infiltration into tumors, the formation of functional immunological synapses, and the destruction of malignant cells, while also exploiting LFA-1 to promote metastasis. Understanding these evasion mechanisms is crucial for developing therapeutic strategies that enhance leukocyte infiltration and function within the TME. This section will delve into how tumors manipulate LFA-1, focusing on its implications for cancer progression.

### 4.1 Strategy I: Alterations in LFA-1-mediated migration and infiltration of leukocytes into the tumor microenvironment

Leukocyte trafficking into the TME relies heavily on cell movement toward chemokine gradients and the use of integrins like LFA-1 for adhesion to vasculature and subsequent extravasation into the tumor (43, 97). However, there is evidence that tumors can disrupt these processes. For instance, non-inflammatory tumors with inactivated LFA-1 exhibit impaired T cell priming and reduced recruitment into the TME (98, 99). Although the exact mechanisms of LFA-1 inactivation are not fully understood, these tumors are known to release chemokines such as vascular endothelial growth factor (VEGF), which suppress the expression of endothelial ICAM-1, a crucial ligand for LFA-1 (100–102). This suppression hinders LFA-1-mediated leukocyte recruitment into the TME. Moreover, reduced secretion of chemokines like CXCL12 further impairs T cell infiltration by decreasing the inside-out signaling required for effective LFA-1 activation (98). Normally, these chemokines promote LFA-1 expression on the cell surface and enhance the ability of T cells to infiltrate the TME (98). A recent study on human melanoma patients revealed that tumors secrete the cytokine growth differentiation factor 15 (GDF-15), which hinders LFA-1-mediated adhesion of T cells to activated endothelial cells—a crucial step for T cell extravasation (103). GDF-15 does this by curtailing the interaction of LFA-1 with the actin cytoskeleton via the protein talin. The study also emphasized the significance of LFA-1 in the success of immunotherapy, showing that elevated serum GDF-15 levels in cancer patients were linked to poor outcomes following PD-1 therapy, and that inhibiting GDF-15 improved T cell recruitment and enhanced therapeutic efficacy (103).

The complement receptor C3aR has also been shown to interfere with LFA-1 activation in T cells and other leukocytes, disrupting their trafficking to tissues (104). In line with this, research by Nandagopal et al. revealed that high levels of the inflammatory factor C3a, released within the TME, bind to C3aR, causing the accumulation of high-affinity LFA-1 at the uropod (tail region) of NK cells (105). This suboptimally increases NK cell adhesion to the endothelium, thereby preventing effective infiltration into the TME (105). Also, impaired LFA-1 function in non-classical monocytes results in inefficient killing of metastatic tumor cells during vasculature patrolling (86). These monocytes fail to express Kindlin-3, a key intermediary molecule required for LFA-1 activation, leading to dysfunctional LFA-1 signaling. Consequently, the weakened LFA-1:ICAM-1 interactions cause the monocytes to ‘slip’ rather than firmly adhere to the endothelium, reducing their effectiveness in infiltrating and killing metastatic cells (86).

Regulating cell adhesion and motility is a complex process that involves the intracellular trafficking of integrins to and from adhesion sites, particularly in fast-moving cells like leukocytes (84, 106). In CLL, T cell movement is disrupted due to impaired LFA-1 resulting from continuous direct contact with the tumor cells. Although the precise receptors on T cells and ligands on CLL tumors involved in transmitting these immunosuppressive signals are not fully understood, this contact alters Rho GTPase signaling in T cells by downregulating RhoA and Rac1 and upregulating Cdc42, leading to defective T cell adhesion and motility (84). Further research is needed to fully understand how these impaired T cells migrate into secondary solid tumor niches and whether this process is equally impaired in CLL patients.

### 4.2 Strategy II: Disruptions in LFA-1 functionality in leukocytes within the tumor microenvironment

After infiltrating the tumor microenvironment, leukocytes are met with a barrage of dysregulating factors from the tumor that impair their ability to effectively use LFA-1 for immunological synapse formation and cytotoxic functions. These disruptions occur through multiple mechanisms, including altered LFA-1 activation and clustering, impaired shifting of LFA-1 to its high affinity conformation, and changes in the mechanical properties of the tumor cell surface (46, 47, 107, 108). Collectively, these factors significantly hinder the leukocyte’s capacity to engage and destroy tumor cells via LFA-1 within the TME.

#### 4.2.1 Impairment of LFA-1 activation and clustering

LFA-1 clustering, and activation are crucial for stabilizing interactions between T cells and their target cells (46, 84, 109, 110). A recent ex vivo study by Lacouture et al. demonstrated that human CD8+ T cell activation and degranulation are regulated by the availability of discrete LFA-1 nanoclusters within the cytolytic synapse with P815 mastocytoma target cells (109). However, certain solid tumors can impair LFA-1 activation in cytotoxic T lymphocytes by releasing specific secretory factors (47, 103). One such factor is galectin, which has been shown to coat tumor-infiltrating CD8+ T cells, disrupting the lateral diffusion, recruitment, and activation of LFA-1 at the immune synapse (47). These galectin-covered T cells exhibit defective actin reorganization and reduced adhesion to LFA-1’s ligand, ICAM-1, compromising their ability to secrete cytokines at the synaptic interface. Markedly, the removal of galectin from the surface of these T cells alleviates such defects and restores proper synaptic function (47).

A study by Ramsay et al. found that healthy CD8+ and CD4+ T cells cocultured with CLL B cells exhibited significantly reduced LFA-1 clustering at the immunological synapse compared to those cocultured with healthy B cells (46). This reduction in clustering was associated with a marked decrease in the active form of LFA-1 and impaired recruitment of the tyrosine kinase Lck to the T cell synapse, leading to altered F-actin dynamics (46). Similar LFA-1 impairments have also been observed in T cells isolated from CLL patients (46, 111, 112), highlighting a key mechanism by which CLL cells evade immunosurveillance.

In advanced stages of solid tumors, interleukin-2 (IL-2) cytokine levels are substantially reduced, which diminishes the effectiveness of anti–programmed death 1 (anti-PD-1) antibody therapy (113). IL-2 is crucial for activating STAT5, which in turn enhances LFA-1 activation and expression (62, 113). This further aids in the formation of immunological synapses necessary for effective CD8+ T cell antitumor responses. To address this issue, studies have shown that adoptive transfer therapy—where CD8+ T cells with elevated LFA-1 expression are reinfused into mice—can counteract the reduced IL-2 levels. Enhancing LFA-1 expression or IL-2/STAT5 signaling pathways boosts antitumor efficacy in CD8+ T cells and improves the effectiveness of PD-1 blockade therapy, benefiting both early and late-stage tumors (113).

#### 4.2.2 Impairment of LFA-1’s high affinity conformation

The extended high-affinity conformation of LFA-1 is essential for forming mature immunological synapses between T cells or NK cells and their targets. Blockade of high-affinity LFA-1 in human CD8+ T cells has been shown ex vivo to significantly impair their adhesion to and cytotoxic activity against P815 mastocytoma target cells (109). In a study by Weulersse et al., defective activation of LFA-1 into its high-affinity conformation was identified as a key factor impairing CD8+ T cell responses across several human malignancies, including multiple myeloma, lung adenocarcinoma, breast carcinoma, and ovarian carcinoma (82). By combining experimental approaches using human and mouse samples, this impairment was linked to the loss of CD226—a critical coreceptor that enhances LFA-1 activation—in tumor-infiltrating CD8+ T cells. This loss was driven by the transcription factor Eomes; however, the exact signaling pathways involved in the Eomes-CD226-LFA-1 axis remain an area of active investigation. The resulting defect in LFA-1 activation was associated with CD8+ T cell hyporesponsiveness, evidenced by diminished production of key anti-tumor mediators such as TNF-α and IFN-γ (82).

Extracellular divalent ions such as magnesium (Mg²^+^) are crucial in regulating LFA-1’s conformational dynamics. In CD8+ T cells, Mg²^+^ induces a high-affinity conformational change in LFA-1, enhancing calcium flux and promoting signal transduction, which supports immune synapse formation and cytotoxicity (75). Moreover, in a study by Van Kooyk et al., using the human CD4+ T cell clone JS136 and peripheral blood T lymphocytes, Mg²^+^ regulated LFA-1’s affinity for ICAM-1, while calcium (Ca²^+^) modulated its clustering and surface distribution, highlighting the distinct roles of these divalent ions in T cell adhesion (110). Hypomagnesemia, a condition characterized by low serum magnesium levels, is commonly observed in inflammatory conditions, including cancer (107). Additionally, chronic hypomagnesemia can predispose individuals to cancer (107, 114). Alterations in ion homeostasis within the TME are common in many malignancies, and tumors may outcompete immune cells for essential micronutrients, including ions like Mg²^+^, due to their altered cellular metabolism and rapid division (115, 116). In this context, depletion or reduction of divalent ions in the TME could potentially impair LFA-1’s high-affinity conformation in leukocytes, contributing to tumor immune evasion. However, whether primary tumor development induces hypomagnesemia leading to immune escape, or whether it results from other underlying chronic inflammatory factors, remains a complex research question with significant implications for translational cancer research. Nonetheless, Nasulewicz et al. previously observed that metastatic dissemination of lung carcinoma cells was heightened in mice fed a low-magnesium diet (117). More recently, Lötscher et al. demonstrated that extracellular Mg²^+^ is sensed through LFA-1 and is required for LFA-1-mediated activation and antitumor effector functions of CD8+ T cells (75). They also found that hypomagnesemia in B cell lymphoma patients was associated with increased disease progression and worse outcomes following CAR T cell therapy or immune checkpoint inhibitor treatment (75).

#### 4.2.3 Alterations in tumor cell surface mechanics

Recent cellular biophysical assays by Wang et al. highlighted the importance of mechanical forces in LFA-1 function by demonstrating that regions of active force exertion through LFA-1, within the immune synapse are where lytic granule exocytosis occurs in CD8+ T cells (118). Disrupting these forces, such as by depleting the adaptor molecule talin, significantly reduces CTL cytotoxicity (118). Cancer cells, particularly those in metastatic breast, glioma, or ovarian cancer, often develop softer mechanical properties compared to their non-malignant counterparts (119–122). This reduction in rigidity may play a key role in immune evasion. Supporting this, an *in vitro* study using both mouse and human tumor cell lines from melanoma, colon cancer, breast cancer, and hepatocellular carcinoma revealed that tumor-repopulating cells evade cytolytic CD8+ T-cell killing by exploiting their mechanical softness (123). This softness hinders the formation of perforin pores at the immune synapse, thereby impairing effective T-cell-mediated cytotoxicity. For NK and T cells to effectively form LFA-1-mediated immunological synapses and spread symmetrically around cancer cells, they require a gradual, continuous flow of actin and proper polarization of the microtubule-organizing center (108, 124). These processes rely on the cancer cell surface maintaining optimal stiffness to allow efficient mechanical forces through LFA-1 (108).

In a similar manner, NK cells may fail to respond effectively to softer targets, leading to reduced production of proinflammatory cytokines such as IFN-γ and TNF-α. Using cell-sized alginate beads of varying stiffness as target models, research by Friedmann et al. showed that NK cell activation, including degranulation and cytokine secretion, is enhanced on stiffer targets through LFA-1 (125). This increased stiffness promoted better cell spreading, microtubule-organizing center polarization, and efficient lytic granule delivery at the immune synapse (125). Conversely, interactions with softer targets led to unstable synapses and impaired NK cell cytotoxicity. These findings emphasize the critical role of mechanical tension at the interface between NK cells and their targets in facilitating immune synapse formation and function. The results highlight how changes in cell or tissue stiffness—such as tumor cells becoming softer—could potentially serve as a novel immune evasion strategy in disease contexts. However, further research involving actual tumor-NK cell interactions is needed to confirm and expand on these observations.

### 4.3 Strategy III: LFA-1-mediated tumor invasiveness

In solid tumors, metastatic extravasation involves increased invasiveness through adhesion and transendothelial migration of disseminated cancer cells (126). Tumor-associated leukocytes, such as pro-tumorigenic macrophages, can exacerbate this process. For example, in nasopharyngeal carcinoma, tumor-associated macrophages (TAMs) interact with interferon-stimulated gene 15 (ISG15) via LFA-1 (127). This interaction triggers SRC family kinase (SFK) signaling in the TAMs, leading to the secretion of CCL18 into the TME, which plays a crucial role in promoting tumor cell dissemination (127). Equally, tumor-associated neutrophils (TANs) are known to promote tumor growth and angiogenesis in breast cancer. A study using murine models showed that estradiol increased pro-tumorigenic neutrophil recruitment to the tumor site, in a process facilitated by the overexpression of TGFβ1 and LFA-1 (128).

Microglia, the resident immune cells in the brain that express LFA-1, also play a crucial role in the TME, contributing to tumorigenesis and neoplastic cell growth in low-grade gliomas (LGGs) (129). Although the exact mechanism by which LFA-1 interaction mediates metastasis in LGGs is unclear, genetic ablation of CD11a (a subunit of LFA-1) has been shown to reduce microglia infiltration, proliferation, and CCL5 secretion, thereby decreasing the likelihood of LGG progression (129).

In CLL, leukemic B cells exhibit enhanced adhesion due to LFA-1 activation, which is triggered by CXCL12, leading to the dissemination of CLL cells to secondary lymphoid organs (130, 131). During the pathogenesis of CLL, CXCL12 activates protein tyrosine kinase (PTK), Bruton’s tyrosine kinase (BTK), and Janus Kinase (JAK), which in turn activate the small GTPase RhoA (132). This signaling cascade shifts LFA-1 from an inactive to a high-affinity conformation, thereby promoting adhesion to ICAM-1 and spreading of CLL B cells into secondary niches (131). Elsewhere, human peripheral blood mononuclear cells (PBMCs) from healthy donors or CLL patients were injected into non-irradiated mice, and three hours later, cells were collected from lymph nodes, bone marrow, and spleen. Using flow cytometry, the study found that CLL B cells had reduced LFA-1 expression, impairing homing to lymph nodes but allowing re-entry into the bone marrow via VLA-4 (83). Both normal and CLL B cells could home to the spleen independently of these integrins, suggesting that integrin targeting may block CLL cells from survival niches in lymph nodes and the bone marrow (83).

T regulatory cells (Tregs) serve as an obstacle to effective antitumor immunity (133), and there is evidence suggesting that LFA-1 may influence tumorigenesis through them. A study using LFA-1 knockout mice demonstrated that the absence of LFA-1 significantly inhibited tumor growth in both subcutaneous melanoma and intestinal adenocarcinoma models (134). This tumor suppression was linked to a substantial decrease in Treg cell numbers in the spleen, blood, and mesenteric lymph nodes. Similarly, treatment of tumor-bearing wild-type mice with the LFA-1 inhibitor BIRT377 reduced both tumor growth and Treg cell accumulation. Moreover, analysis of tumor databases revealed a positive correlation between LFA-1 expression, Treg cell infiltration, and tumor progression. These findings suggest that LFA-1 contributes to a pro-tumorigenic microenvironment by promoting Treg cell-mediated immune suppression, drawing attention to its potential as a therapeutic target for cancer immunotherapy.

Interestingly, certain tumors progress and metastasize by leveraging LFA-1’s interaction with its other ligands, particularly ICAM-3 (135, 136). Shen et al. demonstrated that in human lung carcinoma and breast adenocarcinoma, signaling through ICAM-3 promoted cancer cell stemness (135). Their investigation revealed that ICAM-3 upregulation in these tumors correlated with higher tumor grades and played a critical role in driving metastasis via LFA-1 (136). They further showed that ICAM-3 binds LFA-1 through its extracellular domain and associates with the structural protein ERM, while its intracellular domain links to lamellipodia. This interaction generates mechanical tension, facilitating cell separation and metastasis (136).

ICAM-2 has also been implicated in angiogenesis, as shown in a study using ICAM-2–deficient mice and endothelial cells. The absence of ICAM-2 impaired angiogenesis both *in vitro* and *in vivo* by disrupting homophilic interactions that likely contributed to endothelial tube formation (137). ICAM-2–deficient cells exhibited defective migration and increased apoptosis in response to stressors such as serum deprivation, anti-Fas antibody, or staurosporine, underlining its critical role in supporting endothelial cell function during angiogenesis (137). In contrast to its role in promoting angiogenesis, ICAM-2 expression in neuroblastoma has been linked to a reduced metastatic potential. A study using neuroblastoma cells from mouse models demonstrated that ICAM-2 interacted with α-actinin, which strengthened the membrane-cytoskeleton link (138). This interaction reduced cellular invasiveness and motility, thereby supporting a more favorable clinical outcome, suggesting that ICAM-2 might act as a tumor suppressor in neuroblastoma by limiting metastasis. While both studies show ICAM-2’s involvement in regulation of cellular adhesion and actin dynamics, they highlight its context-dependent functions—supporting tumor suppression in neuroblastoma while aiding endothelial migration and survival in angiogenesis.

Altogether, these studies underscore the dual role of LFA-1 in immunosurveillance. They reveal the intricate dependence of leukocytes on this integrin, while also highlighting how tumors and pro-tumorigenic leukocytes, including Tregs, can exploit LFA-1 and its ligands to drive tumorigenesis and metastasis. This duality poses several challenges in understanding LFA-1 dynamics in tumors and leukocytes and complicates the development of LFA-1-targeting therapeutics in oncology.

## 5 Intervention strategies targeting LFA-1-mediated processes in cancer

Following the previous chapter’s examination of how tumors undermine LFA-1-mediated immune responses, this chapter shifts focus to therapeutic strategies that may potentially counteract these cancer immune evasion tactics. As summarized in **Table 1**, we will explore LFA-1-centered interventions that may potentiate: (i) leukocyte infiltration and activation in the TME, (ii) stable LFA-1-mediated synapse formation between leukocytes and tumors to boost cytokine production and cytotoxicity, and (iii) inhibition of LFA-1-driven tumor progression and metastasis.

**Table 1:** A summary of interventions targeting LFA-1-mediated processes.

Intervention	Type of intervention	Targeted tumor	Mode of action	References
Enhancing infiltration of leukocytes into the tumor microenvironment				
7HP349	Allosteric agonist of integrins	Melanoma	Activates LFA-1 to enhance T cell adhesion and spreading on the endothelium	(98)
BIRT377	Allosteric modulator of LFA-1	Colorectal carcinoma	Blocks high-affinity LFA-1 to ensure optimal NK cell adhesion to the endothelium	(105)
Enhancing stable synapse formation between leukocytes and tumor cells				
Lenalidomide	Immunomodulatory drug	Chronic lymphocytic leukemia	Restores proper T cell LFA-1 signaling and F-actin polymerization at the synapse	(46, 84)
Elotuzumab	Immunostimulatory antibody	Ovarian adenocarcinoma	Upregulates ICAM-1 and LFA-1 expression on NK cells, promoting their conjugation with target cells	(139)
Lenalidomide and Elotuzumab	Immunomodulatory drug and antibody	Multiple myeloma	Upregulates ICAM-1 expression on NK cells and tumors, enhancing their conjugation	(140)
Inhibiting LFA-1-driven tumor progression and cancer metastasis				
A286982	Allosteric inhibitor of LFA-1	Nasopharyngeal carcinoma	Inhibits LFA-1, preventing TAMs from producing the metastasis-promoting chemokine CCL18	(127)
Lifitegrast	Inhibitor of LFA-1/ICAM-3 interaction	Lung carcinoma Breast adenocarcinoma	Prevents LFA-1—ICAM-3 binding, thereby inhibiting LFA-1-mediated tumor migration	(136)

### 5.1 Enhancing LFA-1-mediated leukocyte infiltration into the tumor microenvironment

Increased leukocyte infiltration into tumors has long been linked to improved tumor clearance and better cancer prognosis, a correlation that holds true even following immunotherapy (97, 99, 141, 142). Proof-of-concept studies using mouse models have demonstrated that LFA-1-activating interventions can convert a T cell–exclusionary tumor microenvironment into one enriched with T cells (98). Hickman et al. showed that in melanoma, treatment with 7HP349, a small molecule that activates LFA-1, significantly increased the infiltration of tumor-specific CD8+ T cells into tumors by promoting chemoattraction via CXCL12. Furthermore, when 7HP349 was combined with anti–CTLA-4 therapy, infiltration of CD8+ effector T cells in anti–programmed death 1–resistant (anti–PD-1)-resistant tumors was enhanced, and facilitated tumor regression (98). By activating LFA-1, 7HP349 enhances T cell adhesion to ICAM-1, leading to improved cell spreading and migration from the vasculature into the tumor microenvironment.

In contrast, the drug BIRT377, which inhibits the high-affinity conformation of LFA-1 by blocking domain I in the β2 subunit, enhances NK cell infiltration into colorectal carcinoma and drives tumor regression in mice models (105). This mechanism is particularly important because, as discussed in the previous chapter, Nandagopal et al. demonstrated that C3a/C3aR signaling induces high-affinity LFA-1 in the uropods of NK cells, leading to reduced tumor infiltration, likely due to NK cells becoming trapped on the endothelium (105). By preventing this high-affinity LFA-1 state, BIRT377 effectively reduces excessive adhesion of NK cells to the endothelium and supports them to migrate into the tumor microenvironment.

### 5.2 Enhancing LFA-1-dependent stable synapse formation between leukocytes and tumors to promote cytokine production and cytotoxicity

The immunomodulatory drug lenalidomide has been shown to restore impaired immune synapse formation between T cells and CLL B cells, as demonstrated in *in vitro* studies using human samples (46). These defective synapses are primarily due to disruptions in the localization of signaling molecules, including the clustering of LFA-1 at the synapse, which impairs F-actin remodeling—a critical process for the formation of mature synapses (46). Lenalidomide normalizes Rho GTPase signaling, thereby restoring proper downstream signaling of LFA-1, which facilitates effective actin dynamics and the formation of functional immunological synapses (46, 84).

Elsewhere, treatment with the monoclonal antibody elotuzumab, which targets signaling lymphocytic activation molecule family member 7 (SLAMF7) on tumor cells, was shown to enhance conjugation between NK cells and ovarian adenocarcinoma target cells in cell culture (139). This was correlated with upregulated expression of active LFA-1 and ICAM-1 on NK cells, potentially accounting for the increased conjugation. Moreover, there was a notable elevation in the levels of the activating receptor NKG2D on NK cells. These changes collectively facilitated improved NK cell adhesion and cytotoxicity against tumor targets (139). On top of that, combining elotuzumab with lenalidomide significantly increased ICAM-1 expression in both NK cells and multiple myeloma cells, surpassing the effects of either treatment alone (140). This elevated ICAM-1 expression was associated with the formation of stable contacts with tumor cells, leading to enhanced NK cell-mediated cytotoxicity. Overall, these reports emphasize the fundamental role of ICAM-1/LFA-1 interactions in leukocyte-driven tumoricidal processes and demonstrate how LFA-1 functions can be enhanced through combinatorial therapy.

### 5.3 Inhibiting LFA-1-driven tumor progression and cancer metastasis

As discussed in the previous chapter, LFA-1 can adversely influence tumor progression through its interaction with interferon-stimulated gene 15 (ISG15), which activates tumor-associated macrophages (TAMs) (127). These macrophages subsequently release CCL18, a promiscuous chemokine known to drive tumor progression and metastasis (127). In this regard, Chen et al. demonstrated using *in vitro* assays and a murine model that inhibiting LFA-1 with the small allosteric inhibitor A286982 effectively blocked CCL18 secretion from macrophages. Additionally, the increased migration of nasopharyngeal carcinoma cells, induced by CCL18 from the supernatants of ISG15-treated macrophages, was significantly reduced following treatment with this LFA-1 inhibitor (127).

In human lung carcinoma and breast adenocarcinoma, tumor progression and metastasis have been linked to the interaction between ICAM-3 and LFA-1 (135, 136). Lifitegrast, a drug that disrupts the binding between LFA-1 and ICAM-3, was shown through *in vitro* assays and mouse models to inhibit cell migration in both tumor cell lines (136). Furthermore, combining Lifitegrast with an LFA-1 antibody resulted in a significant reduction in tumor cell migration (136).

Taken together, these studies, primarily based on *in vitro* experiments and *in vivo* work in murine models, highlight the critical role of targeting ICAM-1/LFA-1 interactions between leukocytes and tumors for therapeutic purposes. This approach can either enhance leukocyte-mediated tumoricidal activity or, conversely, inhibit LFA-1-driven tumorigenesis and metastasis. Moreover, the findings suggest that in certain cancers, LFA-1 functionality can be optimized through combinatorial therapies, offering a more effective treatment strategy. Nonetheless, to fully realize the clinical potential of these approaches, further studies are essential to validate their utility in clinical settings.

## 6 Conclusions and remarks

In summary, we have explored the role of LFA-1 in promoting immunosurveillance and how tumors subvert these processes by altering LFA-1 functions. We identified three strategies through which tumors can escape immune surveillance via LFA-1-mediated processes: (I) alterations in LFA-1-mediated leukocyte infiltration into the tumor, (II) disruptions in LFA-1 functionality within the tumor, and (III) LFA-1-driven tumor invasiveness. Finally, we reviewed various interventions aimed at overcoming these challenges and emphasized the need for further studies beyond *in vitro* and animal models.

Developing LFA-1-targeting cancer therapies remains complex due to the duality of LFA-1’s roles—beneficial for immune defense but also exploitable by cancer cells during tumorigenesis. This duality is highly context-dependent, and further research is needed to fully understand where LFA-1 can be most effectively leveraged. Therapeutic targeting of LFA-1 requires balancing its pro-tumor activity with its essential role in immune function. Focusing on LFA-1 signaling pathways rather than the protein itself may be promising, though these pathways are often redundant and intertwined with other cellular receptors, adding complexity.

Future research should focus on better understanding how tumors impair immune cells within the TME through LFA-1 and the associated signaling pathways. A deeper understanding of these mechanisms will enable the rational design of LFA-1-targeted interventions. For example, exploring negative regulatory pathways through LFA-1 in tumor-contacted leukocytes, along with the specific molecules involved, may uncover new molecular targets for future therapies.
